# Levofloxacin-Proliposomes: Opportunities for Use in Lung Tuberculosis

**DOI:** 10.3390/pharmaceutics4030385

**Published:** 2012-08-13

**Authors:** Wipaporn Rojanarat, Titpawan Nakpheng, Ekawat Thawithong, Niracha Yanyium, Teerapol Srichana

**Affiliations:** 1 Department of Pharmaceutical Technology, Faculty of Pharmaceutical Sciences, Prince of Songkla University, Hat Yai, Songkhla 90112, Thailand; Email: wipaporn_oil@yahoo.com; 2 Drug Delivery System Excellence Center, Faculty of Pharmaceutical Sciences, Prince of Songkla University, Hat Yai, Songkhla 90112, Thailand; Email: titpawan@gmail.com (T.N.); ekawat281@gmail.com (E.T.); 3 Department of Pharmacology, Faculty of Science, Prince of Songkla University, Hat Yai, Songkhla 90112, Thailand; Email: niracha.y@psu.ac.th

**Keywords:** dry powder inhaler, immunological response, levofloxacin, proliposome, tuberculosis

## Abstract

Levofloxacin (LEV) is a relatively new-generation fluoroquinolone antibiotic that has good activity against *Mycobacterium tuberculosis*. The aims of this study were to develop and evaluate LEV-proliposomes in a dry powder aerosol form for pulmonary delivery. LEV-proliposomes containing LEV, soybean phosphatidylcholine, cholesterol and porous mannitol were prepared by a spray drying technique. The physicochemical properties of LEV-proliposomes were determined using a cascade impactor, X-ray diffraction (XRD), differential scanning calorimetry (DSC) and Fourier transform infrared spectroscopy (FT-IR). The toxicity of proliposomes to respiratory-associated cell lines and its potential to provoke immunological responses from alveolar macrophages (AMs) were evaluated. Antimycobacterial activity using flow cytometry and an *in vivo* repeated dose toxicity test in rats were carried out. LEV-proliposomes were successfully prepared with mass median aerodynamic diameters of 4.15–4.44 μm and with fine particle fractions (aerosolized particles of less than 4.4 µm) of 13%–38% at 60 L/min. LEV-proliposomes were less toxic to respiratory-associated cells than LEV, and did not activate AMs to produce inflammatory mediators that included interleukin-1β (IL-1β), tumor necrosis factor-α (TNF-α), and nitric oxide. The minimum inhibitory concentration (MIC) against *M. bovis* of LEV and LEV-proliposomes containing LEV 10% were 1 and 0.5 µg/mL, respectively. The efficacy of LEV-proliposomes against *M. bovis* was significantly higher than that of free LEV (*p* < 0.05). The efficacy of the LEV-proliposomes against *M. tuberculosis* was equal to that of the free LEV (MIC = 0.195 µg/mL). In a repeated dose toxicity study in rats, renal and liver toxicity was not observed. LEV-proliposomes should now be tested as an alternative formulation for delivering LEV to the lower airways.

## 1. Introduction

Tuberculosis (TB) is a contagious disease that has caused many problems for developing countries. Poverty, immigration and the rapid spread of acquired immunodeficiency syndrome (AIDS) have significantly reduced the effectiveness of controlling the disease [1]. Das and Horton (2010) reported that globally, 2 million TB patients die every year [2]. About 20%–25% of AIDS patients are infected with TB (pulmonary and extra-pulmonary TB infections), so TB is perhaps the most important opportunistic disease associated with AIDS [3]. Therefore control of TB is complicated. Moreover, there are increasing risks of infection by multiple drug resistance-tuberculosis (MDR-TB) strains, resistant to at least two first line medication drugs. Nowadays, antituberculosis drugs are divided into two groups; those used for first and second line medication. The first line medication drugs such as isoniazid, rifampicin, pyrazinamide, ethambutol, streptomycin have high efficacy. The second line medication drugs are ethionamide, cycloserine, para-amino-salicylic acid (PAS), capreomycin, and fluoroquinolones, generally with a lower efficacy, and some have debilitating side effects and are expensive but can be useful in cases of MDR-TB patients [4]. Although the first line medication drugs have high efficacy, long-term treatment (at least six months) can result in liver and renal toxicity. However, non-adherence can result in accelerating the production of drug resistant TB especially MDR-TB. Adequate management of MDR-TB is crucial not only for recovery of the individual but also to prevent the acquisition of more resistant mutations, and the spread of these drug-resistant strains between individuals. Extensive drug-resistant TB (XDR-TB) is defined as an MDR-TB with resistance to a fluoroquinolone or at least one second-line injectable agent (amikacin, kanamycin and/or capreomycin). XDR-TB is associated with a high mortality rate, especially among AIDS patients. This is why second line medication drugs have been introduced and the treatment is of longer duration [4,5,6]. 

Levofloxacin (LEV) is a new-generation fluoroquinolone antibiotic. The *in vitro* and *in vivo* activities of LEV against *Mycobacterium tuberculosis* are two to three-fold greater than for ofloxacin [7]. LEV has a minimum inhibitory concentration (MIC) of 1 µg/L against *M. tuberculosis*, while the MIC of ofloxacin and ciprofloxacin was 2 and 4 µg/L [8,9]. 

An LEV inhalation solution (MP-376, Mpex Pharmaceuticals) is a novel formulation for LEV that is currently being evaluated in clinical trials. MP-376 was developed for the management of cystic fibrosis (CF) patients with chronic respiratory infections due to *Pseudomonas aeruginosa* [10,11,12]. Following administration of MP-376 by nebulizer, high LEV concentrations were achieved in the sputum of CF patients, resulting in a high bactericidal activity [11]. Nebulizers are used mostly only in hospitals for management of chronic disease because they are inconvenient to use. Administration requires a long period of time (0.5–1 h) and the dose delivery is unpredictable. Dry powder inhalers containing a solid drug suspended in a dry powder mix that is fluidized when the patient inhales are portable forms that deliver more predictable doses with ease [13]. 

In this work, LEV-proliposomes for use with dry powder inhalers were prepared by a spray drying method using porous mannitol as the proliposome core carrier that has a low density and high porosity. This study was different from our previous work that employed non-porous microparticles [14]. We expected that porous mannitol would enhance drug delivery to the lung. As the density is decreased the aerodynamic diameter would decrease accordingly so penetration to the alveoli would increase. The morphologies and aerodynamic properties of the LEV-proliposomes were evaluated. Toxicity of the LEV-proliposome formulations on human bronchial epithelial cells, human lung adenocarcinoma cells and alveolar macrophages (AMs) were determined. Phagocytosis of LEV-liposomes by AMs was tested and observed to ensure that reconstituted products can be taken up by AMs. The MIC of the LEV-proliposome dry powder inhaler formulations on both extracellular and intracellular mycobacteria within macrophage cells were determined by a flow cytometric method as previously described [15]. *In vivo* repeated dose toxicities of LEV-proliposome were investigated in male Wistar rats administered with the proliposomes by intratracheal instillation in order to confirm the safety of the formulations.

## 2. Results and Discussion

### 2.1. Morphology of the Microparticles

**Figure 1:**
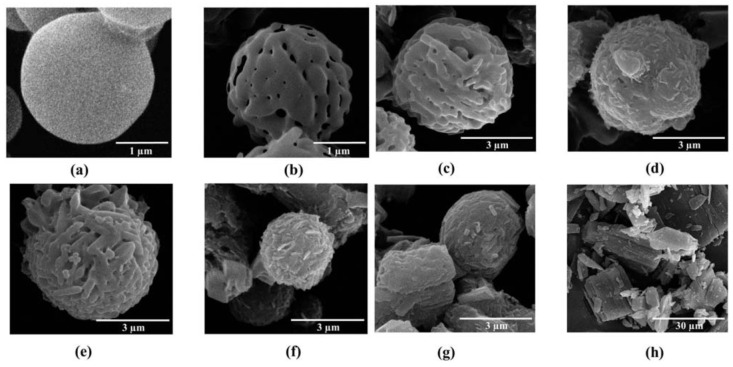
The scanning electron microscope (SEM) images of (**a**) spray dried mannitol (bar =1 μm); (**b**) porous mannitol (bar =1 μm); (**c**–**g**) LEV-proliposome formulation No. 1 to No. 5 (bar =3 μm); (**h**) Levofloxacin (LEV) (bar =30 μm).

Mannitol and ammonium carbonate used as pore forming agents were spray dried together. In the spray drying process, mannitol solution passed through the nozzle as droplets. These droplets rapidly dried and the pore forming agents were volatilized. Spray dried mannitol was prepared for comparison with porous mannitol. The non-porous and porous mannitol microparticles obtained were spheres of around 3 µm (Figure 1a,b). Porous mannitol had a lower density than the non-porous one because of its porosity (data not shown), and this should improve the aerodynamic properties of the mannitol particles. In this study, porous mannitol was used as a core carrier for proliposome, so the proliposomes were expected to have improved aerodynamic properties [16,17].

LEV-proliposomes containing L-α soybean phosphatidylcholine (SPC), cholesterol from lanolin (CH), porous mannitol and LEV were successfully produced using the spray drying technique. The morphology of the LEV-proliposome formulations No. 1 to No. 5 are shown in Figure 1c–g. Spherical microparticles were observed in the formulations No. 1 to No. 3 (Figure 1c–e) with a high (40%–90%) content of porous mannitol, while formulations No. 4–5 (Figure 1f,g) containing porous mannitol produced irregular shapes with some tiny particles (less than 1 µm) or some elongated particles adhering to large aggregated particles. The morphology of the LEV raw material is shown in Figure 1h. 

To prepare the LEV-proliposomes, the lipid part and LEV were dissolved in 95% alcohol. Porous mannitol was then dispersed in that solution. Porous mannitol acted as a proliposome core carrier, LEV could be adsorbed on the carrier and coated with lipid. In this study, LEV-proliposome formulations No. 1 to No. 4 were spherical in shape. This might result from the formulations having sufficient porous mannitol. The formulation No. 5, especially, showed irregular particle shapes with some spheres. It could be that the amount of porous mannitol in formulation No. 5 was not sufficient to produce spherical LEV-proliposome particles. Porous mannitol is an important ingredient for producing spherical particles using the spray drying method. LEV-proliposome formulations were very different in shape from pure LEV (Figure 1h). It seems that the ideal LEV-proliposome formulation should contain not less than 40% porous mannitol to obtain perfect spherical particles. 

### 2.2. *In vitro* Evaluation of the Aerosol Performance of LEV-Proliposome Dry Powder by Cascade Impactor

The percent relative standard deviation of the drug content was less than 6 (Table 1). This value was obtained from the standard deviation divided by the mean of the drug content [18] as it indicated that there was a uniform distribution of the active ingredient throughout the LEV-proliposomes produced using the spray drying process. All formulations showed a content uniformity in the range of 96%–110% of the labeled amount.

LEV-proliposome formulations showed no difference in their mass median aerodynamic diameter (MMAD) (4.15–4.44 μm, *p* > 0.05). MMAD values were correlated with the fine particle fraction (FPF; the fraction of particles smaller than 4.4 µm) in the range of 13%–38% and the emitted dose (ED) value was in the range of 76%–91%. The FPF increased when the MMAD value decreased. LEV-proliposome formulation No. 4 gave a lower MMAD, and a higher FPF, with the highest ED value of 91% among all the formulations. The particles that could reach the lower part of the respiratory tract should be larger than 1 μm and smaller than 5 μm when traveling in the air [16]. From the results, all prepared LEV-proliposome formulations met this requirement. 

**Table 1:** Levofloxacin (LEV)-proliposome formulations and their aerosolization properties (mean ± SD, *n* = 3).

Formulation	Content uniformity (%)	MMAD (μm)	% FPF	% Emitted dose	Encapsulation	size measurement (nm)
**No. 1**	103.5 ± 5.3	4.44 ± 0.26	13.5 ± 1.8	76.3 ± 3.5	23.9 ± 1.5	563.1 ± 18.6
**No. 2**	96.6 ± 2.8	4.33 ± 0.23	22.5 ± 6.0	80.3 ± 6.2	21.9 ± 1.7	672.2 ± 25.7
**No. 3**	109.8 ± 3.8	4.20 ± 0.01	27.2 ± 1.2	81.6 ± 5.1	18.7 ± 1.3	617.9 ± 14.5
**No. 4**	97.4 ± 1.4	4.15 ± 0.03	38.1 ± 4.6	91.3 ± 6.3	16.9 ± 1.5	466.8 ± 9.4
**No. 5**	104.1 ± 3.7	4.17 ± 0.02	28.6 ± 5.0	87.2 ± 6.9	14.9 ± 1.9	1005.3 ± 63.7

LEV-proliposome formulations in this study were prepared using different contents (20%, 40%, 60%, 80% and 90%) of porous mannitol as the core proliposome carrier. From the aerosolization properties of all formulations, the LEV-proliposome formulation No. 4, containing only 40% porous mannitol, had the most suitable aerosolization properties and the particle morphology was spherical in shape. To deliver dry powder particles to the lower part of the respiratory tract, both the formulations and the device factors must be considered.

### 2.3. Encapsulation and Size Measurement after Reconstitution of LEV-Proliposome into a Liposome Suspension

The proliposomes immediately formed a liposomal dispersion on contact with water [19,20]. Liposomes can be used for the encapsulation of hydrophilic and hydrophobic substances [20]. The LEV-proliposome was reconstituted into an LEV-liposome; the vesicle sizes of the LEV-liposomes are shown in Table 1. The sizes of the LEV-liposome had a normal distribution. The mean size ranges were 466–1005 nm from all formulations. Formulation No. 4 gave the lowest vesicle size (466 nm). Macrophage cells normally take up particles that are 200–600 nm in size [21]. This indicates that LEV-liposomes could be phagocytosed. 

The encapsulation efficiency of LEV was in the range of 15%–24% (Table 1). After the LEV-proliposome was reconstituted with distilled water, free LEV would dissolve instantly. Ultracentrifugation was used for separation of the free LEV and LEV-encapsulated liposomes, the liposome was packed into a pellet at the bottom of centrifuge tube. The free LEV in the supernatant was measured. The encapsulation efficiency was calculated from the difference between the LEV content and the free LEV values. Formulation No. 1 had the highest LEV encapsulation, while formulation No. 5 had the lowest. Increasing the drug loading produced a lower encapsulation. Perhaps the lipid amount was not sufficient to encapsulate the drug, so a low LEV loading amount in the formulation gave a high encapsulation efficiency. In the spray-drying process, LEV could be incorporated into the lipid part before the lipid coated onto the porous mannitol particles. An LEV:porous mannitol ratio of 1:9 gave the highest encapsulation efficiency. It might be explained that hydrophobic drugs are normally encapsulated in lipid layers, while hydrophilic drugs are encapsulated inside an aqueous phase [22]. LEV is a hydrophobic drug, so it can be encapsulated only in the lipid layers, thus resulting in low encapsulation. To enhance the encapsulation of a hydrophobic drug in liposomes, encapsulation of the liposomes with cyclodextrin inclusion complexes would increase their water-solubility [23].

### 2.4. X-ray Diffraction (XRD) Measurement of LEV-Proliposomes

**Figure 2:**
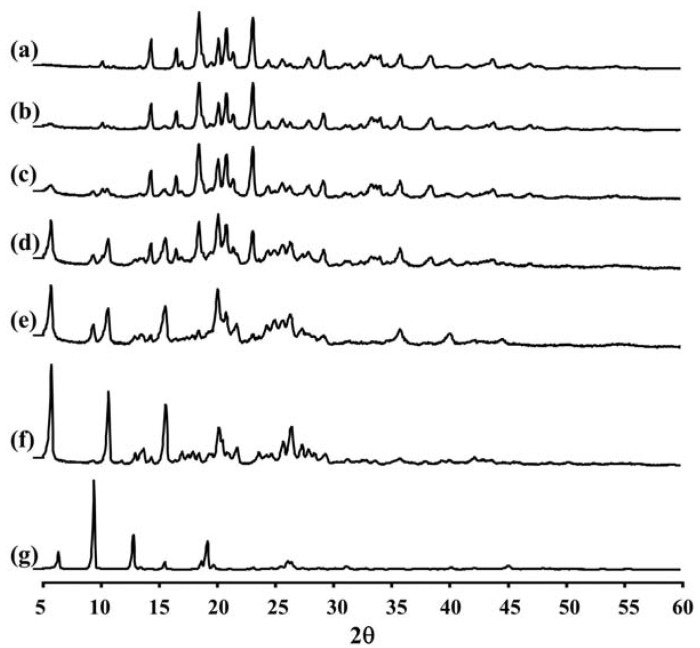
The X-ray diffraction patterns of (**a**) porous mannitol; (**b**–**f**) LEV-proliposome formulation No. 1 to No. 5; (**g**) LEV.

Figure 2 and Table 2 show the XRD pattern and XRD data of the porous mannitol, LEV-proliposome formulations No. 1 to No. 5 and LEV. The porous mannitol diffractogram exhibited sharp reflections at 2*θ* values of 14.58°, 16.76°, 18.70°, 20.38°, 21.06°, 21.67°, 23.35° and 29.43° (Figure 2a). Whereas the diffractogram of LEV showed reflections at 2*θ* values of 6.62°, 9.68°, 13.06°, 15.76°, 18.88°, 19.44°, 26.31° and 26.67° (Figure 2g and Table 2). The sharp reflections of the diffractogram of the porous mannitol and LEV confirm the crystallinity of the ingredients.

**Table 2:** X-ray diffraction peaks (2*θ*) with *d*-spacing in the bracket (Å) of porous mannitol, LEV-proliposomes and LEV.

Material or formulation						
Porous mannitol	No. 1	No. 2	No. 3	No. 4	No. 5	LEV
14.58 (6.08)	14.57 (6.08)	14.57 (6.08)	5.99 (14.75)	5.98 (14.77)	6.02 (14.69)	6.62 (13.34)
16.76 (5.29)	16.76 (5.29)	16.74 (5.30)	10.91 (8.11)	9.62 (9.19)	10.93 (8.09)	9.68 (9.14)
18.70 (4.74)	18.71 (4.74)	18.70 (4.75)	15.85 (5.59)	10.90 (8.12)	13.96 (6.34)	13.06 (6.78)
20.38 (4.36)	20.39 (4.36)	20.36 (4.36)	18.70 (4.75)	15.84 (5.59)	15.87 (5.58)	15.76 (5.62)
21.06 (4.22)	21.06 (4.22)	21.06 (4.22)	20.36 (4.36)	20.32 (4.37)	20.39 (4.35)	18.88 (4.70)
21.67 (4.10)	21.66 (4.10)	21.66 (4.10)	21.04 (4.22)	21.04 (4.22)	20.74 (4.28)	19.44 (4.57)
23.35 (3.81)	23.36 (3.81)	23.34 (3.81)	23.34 (3.81)	25.23 (3.53)	25.96 (3.43)	26.31 (3.39)
29.43 (3.04)	29.43 (3.04)	29.43 (3.04)	26.59 (3.35)	26.66 (3.34)		26.67 (3.39)

LEV-proliposome formulations No. 1 and No. 2 showed intense peaks of crystallinity (Figure 2b,c), similar to that of porous mannitol. For the formulations No. 3, No. 4 and No. 5, new distinct reflections were present at 2*θ* values (calculated *d*_spacing_) of 5.99° (14.75) and 10.91° (8.11) for formulation No. 3, 5.98° (14.77), 10.90° (8.12) and 25.23° (3.53) for formulation No. 4 and 6.02° (14.69), 10.93° (8.09), 13.96° (6.34), 20.74° (4.28) and 25.96° (3.43) for formulation No. 5 (Figure 2d–f and Table 2). 

The X-ray diffraction patterns of the LEV-proliposome formulations No. 3, No. 4 and No. 5 were different from that of porous mannitol and LEV, which may indicate that new crystals or cocrystals had been produced. 

### 2.5. Differential Scanning Calorimetry (DSC) of LEV-Proliposomes

**Figure 3:**
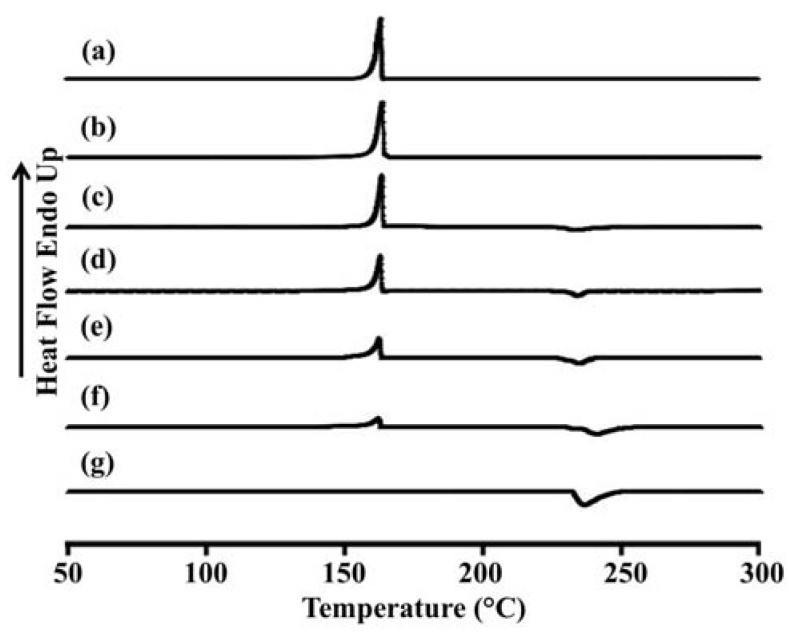
Differential scanning calorimeter thermograms of (**a**) porous mannitol; (**b**–**f**) LEV-proliposome formulation No. 1 to No. 5; (**g**) LEV.

The thermal behavior of the LEV-proliposomes in relation to the individual ingredients was evaluated using DSC experiments. Figure 3 and Table 3 show the DSC thermograms and thermal data for porous mannitol, LEV-proliposome formulation No. 1 to No. 5 and LEV. Porous mannitol showed an endothermic peak at 163.5 °C and a melting point at 151.8 °C. Its enthalpy (Δ*H*_f_) was 266.5 J/g (Figure 3a and Table 4). LEV displayed an endothermic peak at 236.4 °C, and a melting point at 231.9 °C and Δ*H*_f_ −195.6 J/g (Figure 3g). 

**Table 3:** Differential scanning calorimeter data of porous mannitol, LEV-proliposome formulations and LEV.

Material or formulation	Peak (°C)	Onset (°C)	End (°C)	Peak area (J/g)
Porous mannitol	163.5	151.8	166.5	266.5
No. 1	163.5	150.3	166.0	245.1
No. 2	163.3	151.0	166.4	196.4
	233.3	227.2	240.5	−25.2
No. 3	162.8	145.2	165.4	139.8
	234.0	223.9	241.8	−57.1
No. 4	162.3	146.4	164.9	97.4
	235.4	222.8	242.4	−79.9
No. 5	161.8	150.6	165.1	151.8
	241.2	233.2	250.3	−52.3
Levofloxacin	236.4	231.9	249.5	−195.6

**Table 4:** Specific characteristics of LEV-proliposomes as compared with porous mannitol and LEV from the IR spectra.

Material or formulation	Assignments			
	C=O stretching vibration	O–H in plane vibration	C–F stretching vibration	O–H stretching vibration
Porous mannitol	–	1419	–	1080, 1020
No. 1	1624	1419	–	1080, 1020
No. 2	1715, 1624	1419	–	1080, 1020
No. 3	1715, 1623	1407	–	1081, 1020
No. 4	1715, 1622	–	1242	1085, 1022
No. 5	1714, 1622	–	1241	–
LEV	1725, 1621	–	1241	–

The endothermic peak, melting point and Δ*H*_f_ of LEV-proliposome formulations No. 1 to No. 5 are shown in Figure 3b−f and Table 3. For the LEV-proliposome formulation No. 1, the DSC curve was similar to porous mannitol. There were two peaks in the DSC curves of formulations No. 2 to No. 5; the first peak was an endothermic peak and the second was an exothermic peak. These peaks were from the porous mannitol and LEV peaks, respectively. The endothermic peak tended to decrease when the porous mannitol ratio decreased, while the exothermic peak tended to decrease when the LEV concentration decreased. Both peaks of the LEV-proliposome formulations No. 2 to No. 5 showed a broadening peak with a shift to a lower temperature. This indicated that there was some interaction between the LEV and porous mannitol. These results could also be correlated with the X-ray diffraction patterns of the LEV-proliposome formulation No. 2 to No. 5. 

### 2.6. Fourier Transform Infrared Spectroscopy (FT-IR) of LEV-Proliposomes

**Figure 4:**
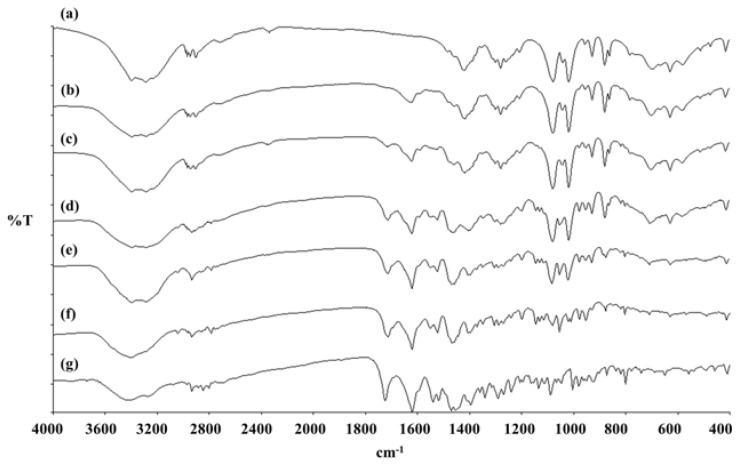
Fourier Transform Infrared Spectroscopy (FT-IR) spectra of (**a**) porous mannitol; (**b**–**f**) LEV-proliposome formulation No. 1 to No. 5; (**g**) LEV.

FT-IR studies were conducted to investigate the relationships between the components of the proliposomes. The mannitol spectrum (Figure 4a) exhibited characteristic bands of O–H in the plane vibration (1419 cm^−1^) and an O–H stretching vibration (1080 and 1019 cm^−1^). The characteristic peaks of LEV (Figure 4g) were seen at 1725 cm^−1^, 1621 cm^−1^ and 1241 cm^−1^ corresponding to C=O stretching (vibration), C=O stretching and C–F stretching (vibration), respectively. The O–H in the plane vibration peak were absent in the formulations No. 4 and No. 5. The C–F stretching vibration disappeared in the formulation No. 1, No. 2 and No. 3. In the formulations No. 1 to No. 5 spectra (Figure 2b–f), the C=O stretching vibration peak shifted (Table 4). This might be due to an interaction between LEV and mannitol. 

From the X-ray diffraction patterns, the DSC data and FT-IR spectra, all data showed that some changes of crystallinity did occur in the LEV-proliposome formulations No. 2 to No. 5. This is an interesting point that will be further investigated.

### 2.7. Cytotoxicity of the LEV-Proliposomes to Respiratory Tract Cells

The viabilities of respiratory associated cells including bronchial epithelial cells, alveolar basal epithelial cells and AMs were evaluated after being challenged with different concentrations of LEV and LEV-proliposomes (Formulation No. 1 and No. 5). In the cytotoxicity study, formulations No. 1 and No. 5 were chosen because of their porous mannitol content. LEV-proliposome formulation No. 1 and No. 5 contained the highest and the lowest amounts of porous mannitol, respectively. Not only did the amount of LEV have an effect, there was also an effect due to the amount of porous mannitol in the formulation on the respiratory cell lines. 

**Figure 5:**
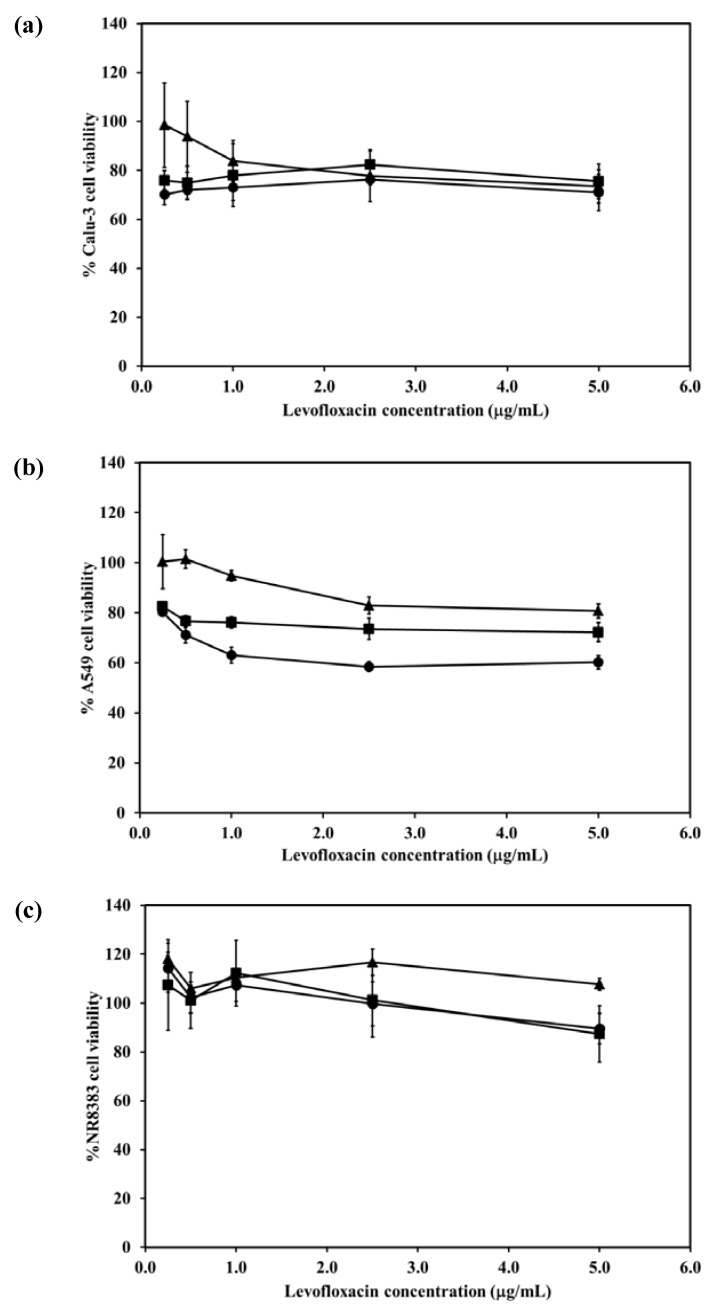
Viability of (**a**) Calu-3; (**b**) A549; (**c**) NR8383 cell lines after exposed to different concentrations of LEV (●); LEV-proliposome formulation No.1(▲);LEV-proliposome formulation No.5 (■) (mean ± SD, *n* ≥ 6).

**Figure 6:**
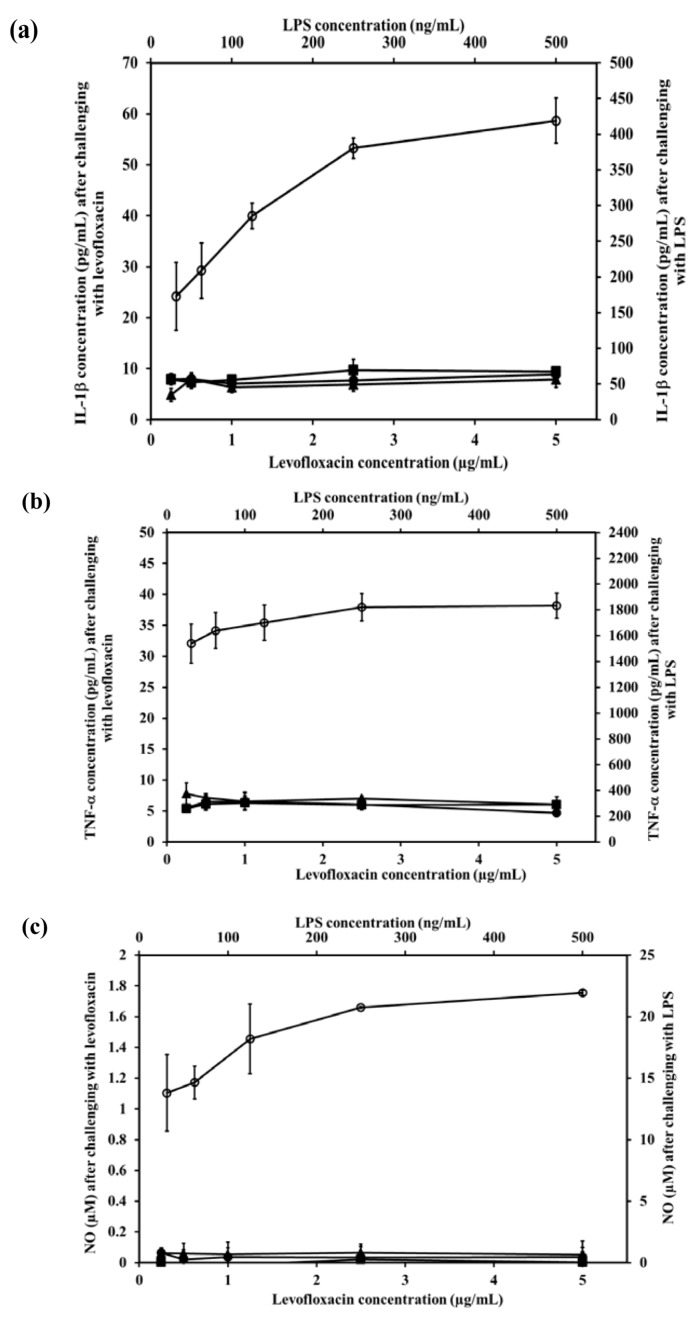
The level of inflammatory cytokine ((**a**) IL-1β and (**b**) TNF-α) and (**c**) nitric oxide produced by NR8383 cell lines after exposure to different concentrations of LEV (●), LEV-proliposome formulation No.1 (▲), LEV-proliposome formulation No.5 (■) and LPS from *E. coli* (**○**) for 24 h (mean ± SD, *n* ≥ 6).

Figure 5a shows the Calu-3 cell viability after exposure to LEV, and LEV-proliposome formulations No. 1 and No. 5 at various concentrations (0.25–5 µg/mL LEV contents). Calu-3 cell viability was more than 80% after being exposed to both LEV-proliposome formulations, while the cell viability was less than 80% when exposed to LEV alone. The concentrations of 0.25–5 µg/mL LEV and LEV-proliposome formulation No. 5 was toxic to A549 cells (cell viability less than 80%), while the LEV-proliposome formulation No. 1 at 0.25 and 0.55 µg/mL LEV was nontoxic to A549 cells (Figure 5b). For the NR8383 cells (Figure 6c), the viability after being exposed to LEV, LEV-proliposome formulation No. 1 and No. 5 at all LEV concentrations (0.25–5 µg/mL LEV content) was not affected. 

This cell viability study showed that all three cultured cell lines were more resistant to the toxic effects of LEV in the form of LEV-proliposome formulations No. 1 and No. 5 than to pure LEV. Hence, proliposome could be used to reduce the drug toxicity to cells in the respiratory tract [24].

### 2.8. Effect of LEV-Proliposome on Production of Interleukin-1β (IL-1β), Tumor Necrosis Factor-α (TNF-α), and Nitric Oxide (NO) by the NR8383 Cell Line

Alveolar macrophage NR8383 cells produce inflammatory mediators including IL-1β, TNF-α and NO after being stimulated. To evaluate the effect of LEV, and LEV-proliposome formulations No. 1 and No. 5 on the production of inflammatory mediator by NR8383 cells, lipopolysaccharide (LPS) from *E. coli* was used as positive control and cell culture media was used as negative control. 

The IL-1β levels produced from NR8383 cells in response to LPS increased significantly (*p* < 0.01). In contrast after stimulation by LEV, LEV-proliposome formulations No.1 and No.5 (0.25–5 µg/mL LEV content) NR8383 cells produced less than 10 pg/mL IL-1β and there were no significant differences among the preparations (*p* > 0.05) (Figure 6a). 

A similar situation was obtained for the generation of TNF-α. Stimulation by 31.25 and 500 ng/mL LPS induced production of 1538 and 1833 pg/mL TNF-α, respectively in NR8383 cells, whereas in response to LEV, LEV-proliposome formulations No. 1 and No. 5 in the range of 0.25–5 µg/mL LEV content, cytokine production was always less than 10 pg/mL (Figure 6b). Again TNF-α production from NR8383 cells responding to LEV, LEV-proliposome formulation No. 1 and No. 5 in the range of 0.25–500 µg/mL as LEV content did not differ (*p* > 0.05).

Nitric oxide produced by NR8383 cells also responded in a similar way. Stimulation by LPS at 31.25 and 500 ng/mL stimulated NR8383 cells to produce NO at 13.8 ± 3.1 and 22.0 ± 0.2 µM, respectively. NO generated from NR8383 cells responding to LEV, LEV-proliposome formulations No. 1 and No. 5 in the range of 0.25–5 µg/mL LEV content was less than 0.2 µM (Figure 6c).

As expected, NR8383 cells produced negligible amounts of IL-1β, TNF-α and NO in response to LEV, LEV-proliposome formulations No. 1 and No. 5 compared to the LPS. The concentration of LPS used to stimulate NR8383 cells to produce the immunological response was 10-times less than the concentration of any LEV-proliposome. LPS activation of NR8383 cells to produce inflammatory cytokines was significantly greater than when it was challenged with LEV, LEV-proliposome formulations No. 1 and No. 5 (*p* < 0.05). LEV itself did not affect cytokine production by AMs [25].

### 2.9. Activity of LEV and LEV-Proliposome against *M. Bovis*

**Figure 7:**
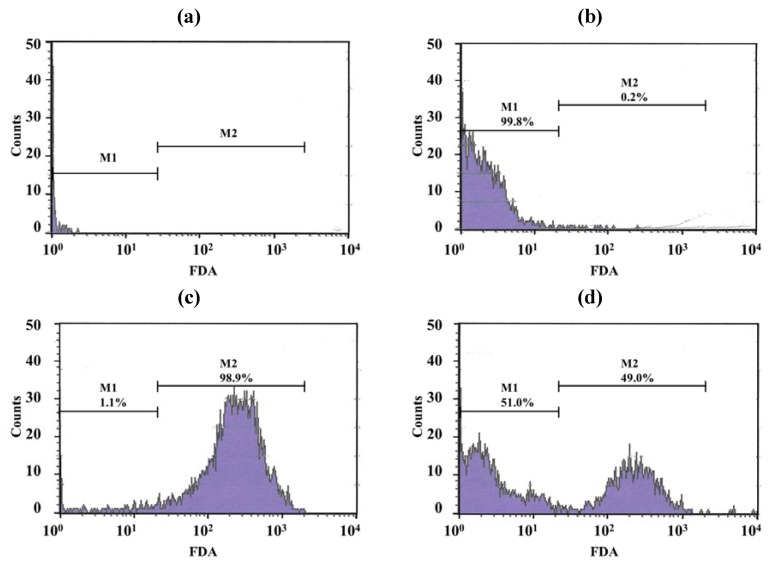
Histograms with histogram markers M1 and M2 of (**a**) M7H9 medium; (**b**) unstained viable *M. bovis*; (**c**) viable *M. bovis* stained with fluorescein diacetate; (**d**) viable *M. bovis* incubated with LEV-proliposome formulation No. 1 at 0.25 μg/mL for 5 days and then stained with fluorescein diacetate (FDA).

Only a few particles were detected in the Middlebrook 7H9 broth (M7H9) medium (Figure 7a) and they did not interfere with the analysis. Unstained viable *M. bovis* were present in the M1 region (Figure 7b) but fluorescein diacetate (FDA)-stained viable *M. bovis* were present in the M2 zone (Figure 7c). The number of *M. bovis* can be calculated from the channel of the event [15]. Figure 7d shows dead cells (in the M1 zone) and viable cells (in the M2 zone) of *M. bovis* incubated with LEV-proliposome formulation No. 1 at 0.25 μg/mL for 5 days and then stained with FDA.

**Figure 8:**
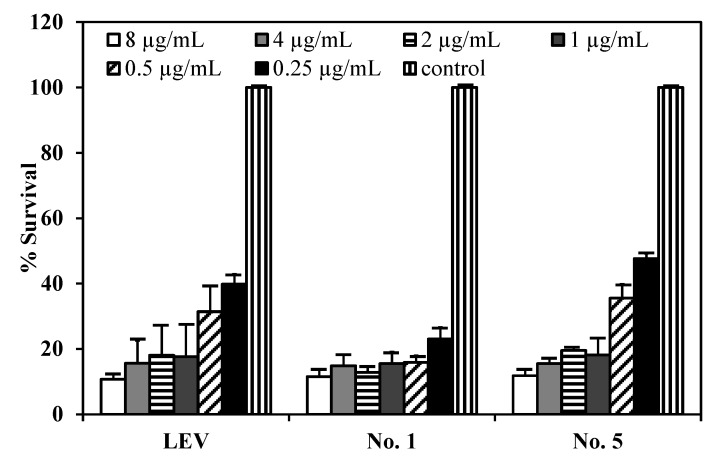
The reduction of *M. bovis* (% survival) after incubation with (**a**) LEV; (**b**) LEV-proliposome formulation No. 1; and (**c**) LEV-proliposome formulation No. 5 at different concentrations for 6 days (Mean ± SD, *n* ≥ 3).

Figure 8 shows the survival of *M. bovis* after incubation with LEV, LEV-proliposome formulation No. 1 and No. 5 at various concentrations. LEV and LEV-proliposome formulation No. 5 showed similar activity against *M. bovis*, while LEV-proliposome formulation No. 1 showed different activity at day 6. At day 1, all samples showed no activity at all concentrations (data not shown). At day 6, viable bacilli were around 10% in most samples treated with LEV at >1 µg/mL either by itself or in liposomes. This indicated that LEV could kill *M. bovis*. The MIC values of levofloxacin against *M. bovis* in this study were 1 µg/mL for LEV and LEV-proliposome formulation No. 5 and 0.5 µg/mL for LEV-proliposome formulation No. 1. These results are similar to those reported by Rastogi and co-workers (1996) [26].

### 2.10. Activity of LEV and LEV-Proliposome against Intracellular *M. bovis* in Macrophage Cells

**Figure 9:**
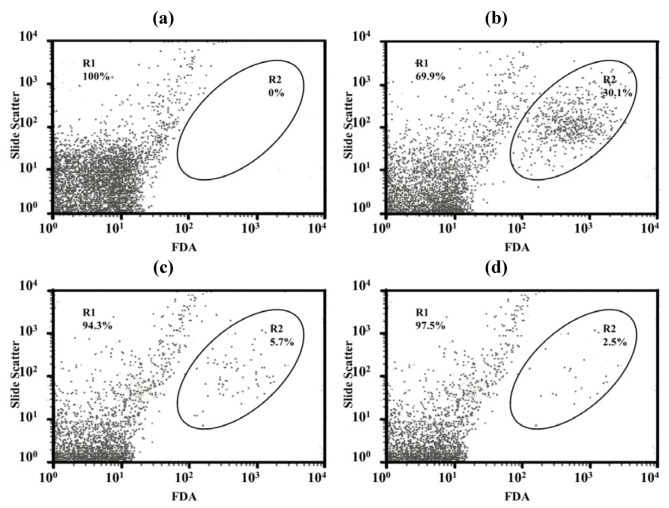
Dot plot analysis with region of (**a**) uninfected macrophage cells; (**b**) viable *M. bovis* intracellular macrophage cells (control) and viable *M. bovis* intracellular macrophage cells incubated with LEV-proliposome formulation No. 1 at 5 μg/mL for (**c**) 5 days; (**d**) 7 days and then stained with FDA.

The number of *M. bovis* present in intracellular macrophage cells was determined using a dot plot analysis after infected macrophage cells were lysed then stained with FDA. Lysed NR8383 cells showed few particles in selected regions (Figure 9a) but macrophage cells within intracellular viable *M. bovis* cells displayed large numbers of particles in the selected region (Figure 9b). The particle counts in the selected region decreased when *M. bovis* infected macrophage cells were incubated with LEV-proliposome formulation No. 1 (5 μg/mL) for 5 days and the numbers decreased further after 7 days (Figure 9c,d).

**Figure 10:**
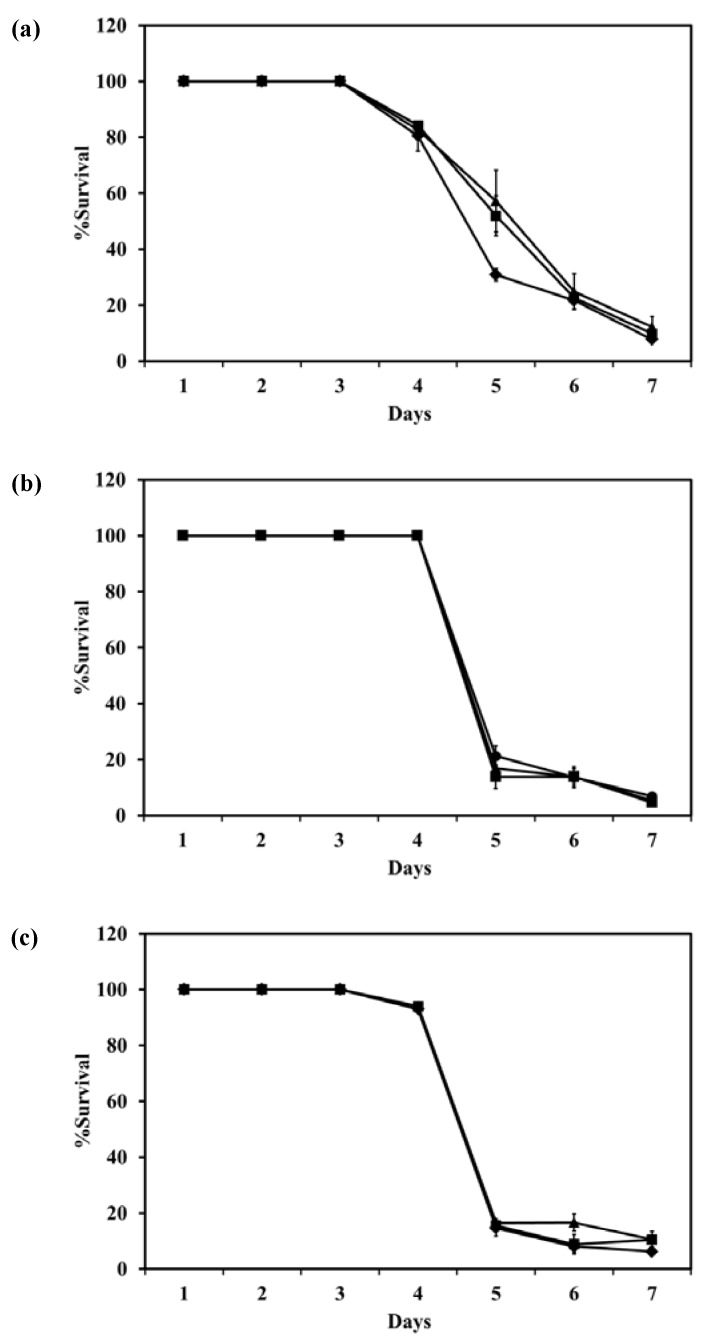
The reduction of intracellular *M. bovis* (% survival) in macrophage cells after incubation with (**a**) LEV; (**b**) LEV-proliposome formulation No. 1; (**c**) LEV-proliposome formulation No. 5 at different concentrations (●) 5; (▲)10; (■)20; (♦)40 µg/mL (Mean ± SD, *n* ≥ 3).

The survival of intracellular *M. bovis* in macrophage cells after incubation with LEV, LEV-proliposome formulations No. 1 or No. 5 at various concentrations is shown in Figure 10. LEV-proliposome formulation No. 1 and No. 5 showed a similar curve of inhibition against *M. bovis* present in NR8383 cells, while LEV itself showed a different curve with longer survival. From the first day to the third day, all samples showed no loss of viability at any concentrations. At day 4, the numbers of viable bacilli decreased after incubation with LEV alone then decreased steadily to <10% at day 7. For the LEV-proliposome formulation No. 1 there was no change from day 3 to day 4, and for the LEV-proliposome formulation No. 5 there was a slight loss of viability of <10%. From day 4 to 5 the cell numbers decreased dramatically by <10% for both proliposome formulations. This again showed that LEV-proliposomes were more effective than LEV in killing *M. bovis* in infected NR8383 cells after incubation for 5–7 days. It is expected that this is due to enhanced transport of LEV into *M. bovis*.

### 2.11. Phagocytosis of Antituberculosis Dry Powder Inhaler Formulation Particles by Macrophage Cells

**Figure 11:**
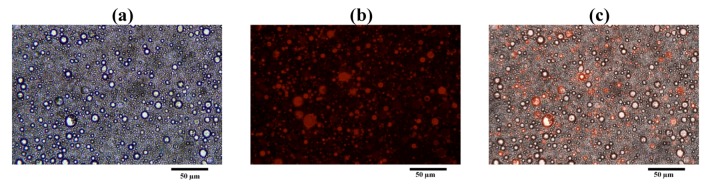
Morphology of LEV-proliposome formulation No.1 reconstituted in distilled water and stained with Lumidot^®^ 640 in different mode; (**a**) bright field; (**b**) fluorescence; (**c**) overlay (bar = 50 µ m).

LEV-proliposome formulation No. 1 reconstituted in distilled water was stained with Lumidot^®^ 640. Figure 11 shows the morphology of LEV-liposome stained with Lumidot^®^ 640, the pictures were taken in bright field (Figure 11a), fluorescence (Figure 11b) and overlay mode (Figure 11c). Vesicle sizes were less than 1 µm. They remained unchanged for up to 24 h after reconstitution. 

**Figure 12:**
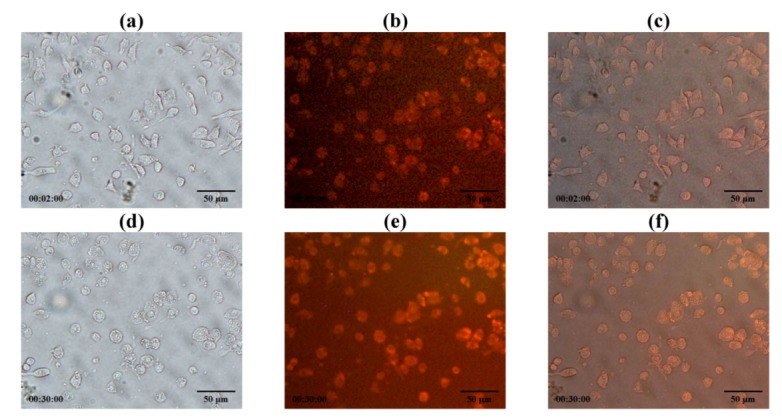
Phagocytosis of NR8383 cells incubated with reconstituted LEV-proliposome formulation No.1 stained with Lumidot^®^ 640 at (**a**–**c**) 2 min; (**d**–**f**) 30 min in different mode; (**a **and **d**) bright field; (**b **and **e**) fluorescence; (**c **and **f**) overlay (bar = 50µm).

LEV-proliposome formulation No. 1 was reconstituted in distilled water and stained with Lumidot^®^ 640 to observe if it was phagocytosed by NR8383 cells at 30 min after incubation of NR8383 cells, preinfected with M bovis, followed by addition of Lumidot stained LEV-liposomes, cells increased in size and shape and the intensity of red color in the overlay mode had significantly increased (Figure 12). This indicated that infected NR8383 cells could take up reconstituted LEV-liposomes stained with Lumidot^®^ 640. The optimum size for efficient phagocytosis was in the range of 200–600 nm [21]. 

### 2.12. Activity of LEV and LEV-Proliposome against *M. Tuberculosis*

The MIC values of LEV, LEV-proliposome formulation No. 1 and No. 5 against *M. tuberculosis* were 0.195 µg/mL. This was lower than found in a previous study [26]. Rastogi and co-workers (1996) reported that the MIC of LEV itself against *M. tuberculosis* was 1 µg/mL [26]. It again showed that LEV-proliposomes had antimicrobial activity against *M. tuberculosis*. The spray drying method was therefore suitable for producing effective LEV-proliposomes. 

### 2.13. *In vivo* Repeated Dose Toxicity Study of the LEV-Proliposomes

The MIC of LEV in LEV-proliposome formulation No. 1 against *Mycobacterium* sp. was 0.5 µg/mL. The amount of the LEV-proliposome formulation was calculated to be 4 mg/kg body weight. In this study, the LEV-proliposome formulations were used at 2000-times the LEV-MIC. *In vivo* repeated dose studies were determined at these extremely high doses for practical reasons to ensure the safety of the formulations. 

**Figure 13:**
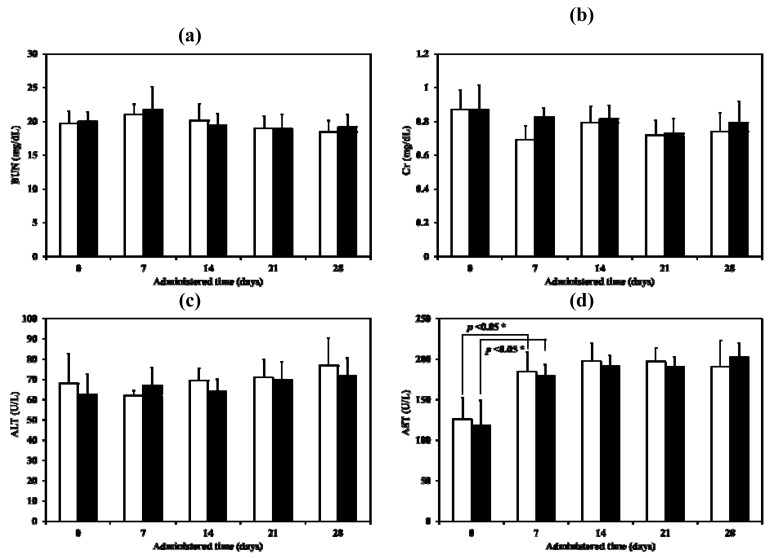
Serum biochemical parameters ((**a**) BUN, (**b**) Cr, (**c**) ALT and (**d**) AST) of control (white column) and LEV-proliposome (black column) treated male Wistar rats in repeated dose toxicity study for 28 days.

For this experiment, the samples were administered by intratracheal instillation at day 0 and then until day 28. There were no significant abnormalities observed in food intake, feces, hair and behaviour in any group of animals during this experimental period. All animals gained weight. No statistical significance in rat body weight during the same time were obtained when compared with the control group (*p* > 0.01). For the serum biochemical parameters, the only significant change observed, in both the control and the test but not between the control and the test, was with the aspartate aminotransferase (AST) after administration for 7 days. (Figure 13a–d). Although AST values did increase, they were still within the normal biochemical ranges for rats (52–224 U/L) [27]. Both ALT and AST in serum become elevated whenever liver cell injury occurs but ALT is the more liver-specific enzyme. Elevations of ALT activity persist longer than those of the AST activity [28]. The change in AST may therefore simply a response to handling. Renal and liver toxicity in the repeated dose toxicity study were not observed when the LEV-proliposome was inhaled.

## 3. Experimental Section

### 3.1. Materials

Mannitol, SPC, and CH were from Fluka (Buchs, Switzerland). Ammonium carbonate was from Ajax Finechem (NSW, Australia). Levofloxacin (LEV) was from Sigma-Aldrich (St. Louis, MO, USA), and dimethyl sulfoxide (DMSO) from Riedel-de Haën (Seelze, Germany). All other reagents were of analytical grade.

All solutions used for the maintenance and culture of human bronchial epithelial cells (Calu-3) and human lung adenocarcinoma cell line (A549) were from Gibco (Grand Island, NY, USA). A sample of 3-(4,5-dimethylthiazol-2-yl)-2,5-diphenyltetrazolium bromide (MTT) and FDA were from Sigma-Aldrich. Quantikine^®^ RTA00 and Quantikine^®^ RLB00 kits for rat TNF-α and IL-1β, were from R & D Systems Inc. (Minneapolis, MN, USA). The BCG vaccine for *Mycobacterium bovis* (*M. bovis*) was supplied by the Queen Saovabha Memorial Institute (Bangkok, Thailand). Middlebrook 7H9 culture medium was obtained from Becton Dickinson and Co. (Franklin Lakes, NJ, USA). *M. tuberculosis* H_37_Ra (ATCC 25177) cells were from the American Type Culture Collection (Rockville, MD, USA) and Alamar blue solution was from Alamar Biosciences/Accumed (Westlake, OH, USA), respectively. Zoletil^®^ 100 was from Virbac Laboratories (Carros, France).

### 3.2. Production of Porous Microparticulate Mannitol

Mannitol (4 g) and ammonium carbonate (1 g) were dissolved in 500 mL of distilled water. The solution was sprayed through a 0.7 mm nozzle using a mini spray dryer B-290 (Büchi, Flawil, Switzerland) at an inlet temperature of 110 °C, an atomizing pressure of 800 kPa, and a feeding rate of 3 mL/min. The porous mannitol was separated and collected by the cyclone and then directed into the collecting chamber. 

### 3.3. Production of LEV-Proliposome by the Spray-Drying Technique

The ingredients of the LEV-proliposome formulations, lipid and powder parts, are shown in Table 5. The weighed lipid part and LEV were dissolved in 100 mL of 95% ethanol to obtain the ethanolic lipid solution containing LEV and further sonicated for 15 min. Porous mannitol was suspended in the solution, and the suspension was again sonicated for 15 min in order to deaggregate porous mannitol particles before the spray drying process began. The suspension was continuously stirred to give a homogenous suspension during spray drying. The inlet temperature was set at 90 °C and the atomizer pressure was 800 kPa, with a feed rate of 3 mL/min. The proliposome powder was obtained from the collecting chamber and kept in a desiccator until used. 

**Table 5:** Formulation ingredients for LEV-proliposomes.

Formulation	Lipid part		Powder part	
	SPC (mmole)	CH (mmole)	Porous mannitol (g)	LEV (g)
No. 1	0.06	0.06	0.9	0.1
No. 2	0.06	0.06	0.8	0.2
No. 3	0.06	0.06	0.6	0.4
No. 4	0.06	0.06	0.4	0.6
No. 5	0.06	0.06	0.2	0.8

### 3.4. Morphology of the Microparticulate Mannitol and LEV-Proliposomes

The surface morphology of spray dried mannitol, porous mannitol, LEV and LEV-proliposomes was examined by scanning electron microscopy (SEM). The samples were dispersed on an aluminum stub and coated with gold using a sputtering technique with a Sputter Coater (SPI supply, USA) in an argon atmosphere for 60 s. The particle morphology was then observed by SEM (FEI Quanta 400 FEG, FEI Company, USA). 

### 3.5. Content Uniformity of LEV in the Proliposome Powder

The LEV-proliposomes powder (10 mg) was randomly sampled and weighed. The powder was suspended in methanol (10 mL) to dissolve the lipid content. Fifteen milliliters of distilled water was added, followed by sonication for 15 min to obtain a clear solution. The LEV content of the clear solution was analyzed by high performance liquid chromatography (HPLC). The HPLC system was equipped with an AS 3000 autosampler, a P1000 pump and a UV 2000 detector (Thermo Fisher Scientific, Waltham, MA, USA). A microbondapak C18 column (Phenomenex, Torrance, CA, USA) (250 × 4 mm i.d., 5 µm) was used as a stationary phase. The mobile phase was phosphate buffer:acetonitrile (85:15 *v*/*v*), running at a flow rate of 1 mL/min. UV detection was set at 288 nm. 

### 3.6. *In vitro* Evaluation of the Aerosol Performance of the LEV-Proliposome Dry Powder by a Cascade Impactor

The LEV-proliposome aerosolized properties were investigated using a delivery device made in-house [29]. The aerosolized parameters of the products, including MMAD, FPF and ED, were examined using an Andersen cascade impactor (ACI) (Andersen Instruments, Atlanta, GA, USA). The ACI was applied with a vacuum pump with a flow rate of 60 L/min for 10 s. Triton X-100 (0.1% *v*/*v*) in methanol was used to rinse the samples deposited on each stage. The LEV content was analyzed by HPLC, as described in the content uniformity section. The cumulative percentage of deposition was calculated to its *Z*-value and plotted against the log of the cutoff diameter for each stage. The MMAD was obtained from the particle diameter at a *Z*-value of zero [30]. The emitted dose is the amount of drug removed from the delivery device. FPF is the fraction of particles smaller than 4.4 µm.

### 3.7. Encapsulation and Size Measurement after Reconstitution of LEV-Proliposome into a Liposome Suspension

To evaluate the percentage of encapsulation, 10 mg of proliposome powder was reconstituted with 4 mL of distilled water, and then centrifuged with an ultracentrifuge (Beckman Coulter Inc., Palo Alto, CA, USA). The centrifugation conditions were 100,000*g* for 20 min at 25 °C [31]. The supernatant was measured for unencapsulated LEV by HPLC, as described in the content uniformity section. The percentage of encapsulation was obtained using the following Equation:

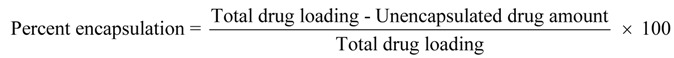
(1)


The size of the liposome after reconstitution was determined using ZetaPALS (Brookhaven, New York, USA) at 25 °C. The LEV-proliposome was reconstituted with milliQ water to obtain nanovesicles while the undissolved particles were removed using centrifugation. The centrifugation conditions were 10,000*g* for 20 min at 25 °C. Sizes were evaluated immediately after receiving the supernatant from the centrifugation process.

### 3.8. XRD Measurements of LEV-Proliposomes

The XRD of the porous mannitol, LEV and LEV-proliposome was carried out with a Siemens D 5000 (Siemens AG, Berlin, Germany) equipped with a diffracted-beam monochromator, using Cu radiation. The samples were spread over a glass sample holder, each in an area of 4 cm^2^ with a depth of 1 mm. The powder surfaces were pressed and smoothed with a glass slide. The diffraction intensity was recorded at an angle of 2*θ* from 5 to 60° with a step size of 0.05° and step time of 1 s. The total time of the diffraction scan was 19 min, and each sample was determined in three separate experiments. The voltage and current generator were set at 40 kV and 30 mA, respectively. The obtained data were analyzed using EVA software. Relative crystallinity was evaluated from the XRD results using the ratio of the intensity of a characteristic crystalline peak to that of the amorphous halo for each powder sample. If the ratio of the peak to the amorphous halo around it decreases, then the crystallinity is proportionately lower [32].

### 3.9. DSC of LEV-Proliposomes

A DSC model 2920 (TA Instruments, Newcastle, DE, USA) was used to examine the interaction of the formulation ingredients in the proliposome powder produced by spray drying. A sample was placed in an aluminum pan, hermetically sealed, and then investigated by DSC from 30 °C to 200 °C at a rate of 10 °C/min. The DSC thermograms were analyzed using the Universal Analysis 2000 program, version 3.4 c.

### 3.10. FT-IR of LEV-Proliposomes

Infrared spectroscopy was used to identify the functional groups of the samples. Dry KBr was carefully mixed with dried sample and pressed into a pellet. The pellet was placed in a magnetic holder. The FT-IR spectra between 4000 and 400 cm^−1^ was examined by an accumulation of 16 scans using a Spectrum One (Perkin-Elmer, MA, USA). 

### 3.11. Cell Cultures

#### 3.11.1. Culture of Human Bronchial Epithelial Cells (Calu-3), Human Lung Adenocarcinoma Cell Line (A549) and Alveolar Macrophage Cell Line NR8383

Human bronchial epithelial cells (Calu-3, ATCC HTB-55, Rockville, MD, USA) were cultured in modified eagles media supplemented with 10% fetal bovine serum (FBS), 50 units/mL penicillin and 50 µg/mL streptomycin. Human lung adenocarcinoma cells (A549, ATCC CCL-185, Rockville, MD, USA) were cultured in Ham’s F12K supplemented with 10% FBS, 50 units/mL penicillin and 50 µg/mL streptomycin. Calu-3 and A549 cells were maintained at 37 °C in a fully humidified atmosphere at 5% CO_2_ in air and the media were changed every 2–3 days. When the cells reached 60%–80% confluency, they were rinsed with phosphate buffered saline solution (PBS). PBS was aspirated, and the cells were covered with 2 mL of trypsin/EDTA solution. The cells were then detached from the plate by the trypsin/EDTA. Cells were centrifuged, resuspended and then transferred to a new culture flask. The medium was replaced with fresh medium two or three times weekly. 

AMs (NR8383, ATCC CRL-2192, Rockville, MD, USA) had been isolated from normal rat lung lavage. NR8383 cells were cultured in F-12 Kaighn’s cell culture medium supplemented with 15% (*v*/*v*) heat-inactivated FBS, 50 units/mL penicillin, 50 μg/mL of streptomycin, and then incubated in a 5% CO_2_ fully humidified atmosphere at 37 °C. The cells were maintained by transferring floating cells to new flasks. Adherent cells might be harvested using a scraper. Upon reseeding, about half of the cells re-attached. The medium was replaced with fresh medium two or three times weekly. 

#### 3.11.2. Determination of Cytotoxicity of LEV-Proliposome to Cells in the Respiratory Tract

Viabilities of Calu-3, A549 and NR8383, respiratory-associated cells, after incubation with LEV, LEV-proliposome formulation No.1 and No.5 were investigated using the MTT assay. This was chosen based on the highest and lowest encapsulation efficiency of LEV. Live mitochondria in living cells transform MTT solution to insoluble purple formazan crystals. Briefly, 100 μL of 1 × 10^5 ^cells/mL was cultured in each well of a 96-well plate and allowed to adhere and grow overnight at 37 °C, in a 5% CO_2_ and a fully humidified atmosphere. One day later, the fresh media (100 μL) was replaced and 100 μL of cell culture media containing either the LEV or LEV-proliposome formulation was added. The cells were then incubated for 24 h. A sterilized stock MTT solution (50 μL of 5 mg/mL in PBS) was added into each well containing 150 μL fresh medium and the cells were incubated for 4 h at 37 °C. Supernatant was then carefully removed and the formazan crystals were dissolved by adding 200 μL of dimethylsulfoxide (DMSO, Riedel-de Haën, Seelze, Germany) and mixed thoroughly. The absorbance was recorded at 570 nm using the microplate reader (Biohit BP 800, Helsinki, Finland). The proportion of viable cells in treated wells was compared to the untreated well (control).

### 3.12. Determination of the AM Response to LEV-Proliposome

#### 3.12.1. Production of Inflammatory Cytokines

Evaluation of the inflammatory cytokine levels (TNF-α and IL-1β) generated from NR8383 cells responding to LEV, LEV-proliposome or LPS from *E. coli*, (positive control) was determined using an enzyme linked immunosorbent assay (ELISA) method. Commercial ELISA kits (Quantikine^®^ RTA00 and Quantikine^® ^RLB00 for rat TNF-α and IL-1β, respectively, R & D systems Inc., MN, USA) were used as described in the product assay procedures. Standards, controls and samples were pipetted into the wells and any rat TNF-α or IL-β present was bound by the immobilized antibody. After washing away any unbound substances, an enzyme-linked polyclonal antibody specific for rat TNF-α or IL-β was added into the wells. Following a wash to remove any unbound antibody-enzyme reagent, a substrate solution was added to the wells. The enzyme reaction yielded a blue product that turned yellow when the stop solution was added. The intensity of the color measured was in proportion to the amount of rat TNF-α or IL-β bound in the initial step. The sample values were then read off a standard curve. The detectable dose of both TNF-α and IL-β was in the range of 12.5–400 pg/mL. 

#### 3.12.2. Nitric Oxide (NO) Assay by the Griess Reaction

NO generated from NR8383 cells after being challenged with LEV, LEV-proliposomes or LPS in a concentration range of 0.25–5 µg/mL for LEV and LEV-priliposome and 25–500 ng/mL for LPS was detected by the Griess reaction. Nitric oxide in the form of nitrite (NO_2_^−^), which is one of the two primary, stable and nonvolatile products of NO was investigated. This measurement relied on the diazotization reaction of the Griess reagent. Griess reagent was prepared by mixing 1% sulfanilamide, 0.1% *N*-(1-naphthyl)-ethylenediamine dihydrochloride and 2.5% phosphoric acid in water. Equal volumes of cell supernatant (100 μL) and Griess reagent (100 μL) were mixed. After mixing for 10 min, the absorbance was examined using a microplate reader at 450 nm. The NO concentration was calculated from a sodium nitrite standard curve [33,34].

### 3.13. Assessments of the Antimycobacterial Activity of LEV and LEV-Proliposome

#### 3.13.1. Culture of *M. Bovis* from BCG Vaccine

The lyophilized BCG vaccine was reconstituted with 1 mL of sterile water for injection. Reconstituted BCG vaccine (200 μL) was grown in 10 mL of Middlebrook 7H9 broth (pH 5.5) containing 0.5% Tween 80% and 10% oleic acid-albumin-dextrose catalase (OADC) enrichment [35]. *M. bovis* was incubated at 37 °C and subcultured every 3 weeks. The *M. bovis* suspension obtained after 3 weeks of culture was used in this experiment.

#### 3.13.2. Determination of MIC against *M. Bovis*

The MICs of LEV and LEV-proliposome (Formulation No. 1 and No. 5) in the LEV concentration range of 0.25–8 µg/mL were evaluated. An inoculum of *M. bovis* was prepared from a suspension of 3-week-old organisms in M7H9 supplemented with Middlebrook OADC enrichment. The bacilli were adjusted with normal saline solution to obtain a turbidity of a McFarland standard of 1 (approximately 3 × 10^8^ CFU/mL). Tenfold serial dilutions (10^−1^, 10^−2^, 10^−3^, and 10^−4^) of the above inoculum suspension was made into the M7H9 medium. Each proliposome concentration (100 μL) was added into wells containing the diluted bacilli suspension (900 μL of 3 × 10^4^ CFU/mL) and incubated at 37 °C. The final concentrations of the LEV and LEV-proliposome were 25, 50, 100, 200 and 400 µg/mL of LEV. Every day until the 6th day, 500 μL of sample was taken from each well and placed in a sterile screw-cap micro-tube containing 500 μL of FDA (500 ng/mL) in PBS at pH 7.4. Samples and FDA were incubated at 37 °C for 30 min before measurements were made using a flow cytometer (FACSCalibur, Becton-Dickinson, California, USA) and CellQuestTM software for data acquisition and analysis [15]. 

#### 3.13.3. Determination of the (MIC) against Intracellular *M. Bovis* in Macrophage Cells

The MIC of LEV and LEV-proliposomes (Formulation No. 1 and No. 5) against intracellular growth of *M. bovis* in NR8383 cells was examined [14]. Prior to infection, NR838 cells were plated at a density of 10^5^ cells/well in 24 well plates and incubated overnight in the CO_2_ incubator to allow the cells to adhere on the well surface. Fresh medium containing 1% fetal bovine serum was replaced in order to reduce cell proliferation and penicillin-streptomycin mixture was excluded to avoid any interference by antibiotics on the following day. *M. bovis* was suspended in F 12 Kaighn’s medium containing 1% FBS and the suspension was dispersed into individual wells at a density of five mycobacterium per macrophage. Infected NR8383 cells were then incubated at 37 °C, in a 5% CO_2_ incubator for 4 h. Following incubation, the supernatant was aspirated and the wells were washed by 3 × 1000 μL with PBS to remove unphagocytosed mycobacteria. Fresh media with and without sample was added into each well. The final concentrations of LEV in the wells were 10, 20 and 40 μg/mL for LEV and LEV-proliposome formulation No. 5 and 5, 10 and 20 μg/mL for LEV-proliposome formulation No. 1, respectively. The infected NR8383 cells were incubated in the 37 °C incubator. Every day for one week, the media was discarded and the wells were carefully washed 3 times with PBS to remove excess sample. Determination of *M. bovis* CFU was conducted by lysing NR8383 cells with 0.125% sodium dodecyl sulfate (SDS) in PBS (500 μL) (*w*/*v*) and incubated at 37 °C for 15 min. The sample was taken from each well and placed in a sterile screw-cap micro-tube containing 500 μL of FDA (500 ng/mL in PBS at pH 7.4). Samples were then incubated at 37 °C for 30 min before being analyzed using a flow cytometer and CellQuestTM software for data acquisition and analysis. 

### 3.14. Phagocytosis by Macrophage Cells Incubated with the Antituberculosis Dry Powder Inhaler Formulation Particles

Proliposome dry powder (Formulation No. 1) was reconstituted, 10 mg/mL in distilled water. Lumidot^®^ 640 (Lumidot^®^, Sigma-Aldrich, St. Louis, USA) 20 μg/mL was added to the liposomes in the ratio 1:2 *v*/*v*. The mixture was sonicated for 3 min and observed with a fluorescence microscope (Olympus, BX61, Olympus, Tokyo, Japan). 

One hundred microlitre of 1 × 10^4 ^cells/mL of NR8383 cells was cultured in each well of a 96-well plate and allowed to adhere and grow overnight at 37 °C, in 5% CO_2_ and a fully humidified atmosphere incubator. Ten microlitre of 1 × 10^6 ^CFU/mL *M. bovis* was added to be phagocytosed by NR8383 cells. Liposome (Formulation No.1) stained with Lumidot^®^ 640 was added. Phagocytosis by NR8383 cells was observed.

### 3.15. Determination of the (MIC) against *M. Tuberculosis*

The antimycobacterial activity of LEV and LEV-proliposomes (Formulation No. 1 and No. 5) against *M. tuberculosis* H37Ra (ATCC 25177) was determined. *M. tuberculosis* was grown in 100 mL of M7H9 broth supplemented with 10% OADC enrichment and 0.05% Tween 80. Antimicrobial susceptibility testing was investigated in a dark room using clear-bottomed, 96-well microplates in order to minimize background fluorescence. The outer wells of the microplates were loaded with sterile water to prevent dehydration in the experimental wells. The first sample was diluted with distilled water; the following two fold dilutions were performed with 0.1 mL of M7H9 (without Tween 80). The final concentration of *M. tuberculosis* was 5 × 10^4^ CFU/mL. A sample without bacteria in the wells was used as a control to detect the autofluorescence of compounds. Plates were incubated at 37 °C. After 4 days of incubation, 20 µL of 10× Alamar Blue solution and 12.5 µL of 20% Tween 80 were added to either test wells (containing bacilli) or control wells (without bacilli), and plates were incubated at 37 °C. Any color change from blue to pink was observed at 12 and 24 h. If a well containing bacterium became pink by 24 h, reagent was added to the entire plate. If the well remained blue, additional bacteria was loaded and tested daily until a color change occurred, at which time reagents were added to all remaining wells. Plates were then incubated at 37 °C, and results were recorded at 24 h after addition of reagent. Visual MICs were defined as the lowest concentration of drug that prevented a color change [36].

### 3.16. *In vivo* Repeated Dose Toxicity Study of the Proliposomes

An *in vivo* repeated dose toxicity study of the LEV-proliposome was performed on male Wistar rats weighing approximately 230–290 g. Animals were obtained from the Southern Laboratory Animal Facility, Prince of Songkla University, Hat Yai, Songkhla, Thailand. They were allowed to adapt to the conditions of the animal house for 1 week before the experiments and kept in an air-conditioned room (22 ± 2 °C) lit for 12 h per day. They were supplied with standard laboratory diets and tap water *ad libitum* during the experiments. All procedures were approved by the Animal Ethics Committees, Prince of Songkla University, Thailand (No. Ref 11/51). Wistar rats were randomly divided into a control and treatment group (*n* = 7 for each group). A blood sample was collected for the pre-treatment samples using the tail vein sampling technique before the porous mannitol or LEV-proliposome was administered. LEV-proliposome formulation No. 1 (4 mg/kg body) was administered by intratracheal instillation at day 0 followed by daily intratracheal inhalation from the 2nd day to the 28th day. The intratracheal instillation and inhalation were modified from a previous study [37]. It was performed during anesthaesia (by Zoletil^®^100 at 40 mg/kg body). A polyethylene tube was inserted through the oropharynx and proliposomes were blown into the tube. For intratracheal inhalation, the rats were allowed to breath into the tube and proliposomes were blown from the other end. During the experiment, blood samples were collected after being first administered for 7, 14, 21 and 28 days. Clinical signs were recorded once daily and body weights were recorded every week during the experiments. The collected blood was centrifuged at 2000 rpm for 10 min to obtain the serum. ALT, AST, BUN and Cr were carried out for determination of liver and renal toxicity. All serum biochemical parameters were analyzed with a Cobas Mira Plus Chemistry Analyzer.

### 3.17. Statistical Analysis

Data, when applicable, were presented as mean ± standard deviation (SD) from at least three samples unless indicated. The data were compared using analysis of variance (ANOVA) followed by a One-Way ANOVA to determine the difference between data sets. All statistical comparisons were calculated using SPSS software version 17 (SPSS Inc., Chicago, IL). A *p* < 0.05 was considered statistically significant.

## 4. Conclusions

LEV-proliposome powders for inhalation were successfully produced using a spray dry method. Their shapes were spherical, except for formulation No. 5 that contained only 10% porous mannitol. The MMAD values of all formulations were in the range of 4.15–4.44 μm, with a FPF of 13%–38%. LEV-proliposome powders reflected the crystallinity of the respective components and varying degrees of interaction with LEV occurred in the formulations depending on the ratios of LEV to mannitol. LEV and LEV-proliposomes were shown to be nontoxic to respiratory-associated cells, and also did not activate AMs to produce inflammatory cytokines and nitric oxide at a level that would cascade to show secondary inflammation. LEV-proliposomes showed good antimycobacterial activity against *M. bovis*, *M. tuberculosis* and intracellular *M. bovis* in macrophage cells. A repeated *in vivo* dose toxicity study of the LEV-proliposomes (4 mg/kg body weight) in Wistar rats, showed no signs of producing renal and liver toxicity. The efficacy of LEV-proliposome inhaled in infected animals or human will be determined in our future work. 
